# Rhabdomyolysis induced by rosuvastatin combined with entecavir: a case report

**DOI:** 10.1186/s12879-022-07254-0

**Published:** 2022-04-11

**Authors:** Wen Wang, Xu Lu, Chengbo Li, Wei Teng, Wei Cui

**Affiliations:** Department of Infectious Diseases, The First Affiliated Hospital of China Medical University, 155 Nanjing North Street, Shenyang, 110001 Liaoning China

**Keywords:** Rhabdomyolysis, Rosuvastatin, Entecavir

## Abstract

**Background:**

Rhabdomyolysis is a serious and potentially life threatening condition that can be caused by drugs. We report a case of acute hepatitis B with rhabdomyolysis after treatment with rosuvastatin and entecavir.

**Case presentation:**

A 72-year-old female was admitted to our hospital due to acute hepatitis B infection. She had taken atorvastatin for 3 months before being admitted to our hospital. After being administered entecavir (ETV) and rosuvastatin to replace atorvastatin, she suffered from muscle pain in both lower limbs and was diagnosed with rhabdomyolysis. After discontinuation of the two drugs, the patient's symptoms subsided and creatine kinase levels returned to normal. We hypothesize that the rhabdomyolysis was caused by the combination of rosuvastatin and ETV.

**Conclusions:**

We suggest that patients who use rosuvastatin and ETV be made aware of the complication of rhabdomyolysis.

## Background

The most important adverse effects associated with statin use are asymptomatic increases in the levels of liver transaminases and myopathy, while rhabdomyolysis is extremely rare. To date, there have been no case reports of ETV-induced rhabdomyolysis. When statins are combined with ETV, the risk of rhabdomyolysis may be increased. Here, we report a case of such an occurrence.

## Case presentation

A 72-year-old female attended our hospital on May 31, 2019 due a “loss of appetite for 20 days, and abdominal pain, yellow skin and yellow eyes for 5 days”. The patient underwent percutaneous coronary intervention (PCI) and stent implantation for coronary heart disease 3 months prior. After the operation, the patient took aspirin, atorvastatin, clopidogrel, benazepril, metoprolol and felodipine, and had no history of blood transfusion or drug abuse. The results of laboratory examinations were as follows: Liver function: alanine aminotransferase (ALT), 1661 U/L (7–40); alkaline phosphatase (ALP), 386 U/L (50–135); gamma glutamyltransferase (GGT), 829 U/L (7–45); total bilirubin (TBIL), 126.1 μmol/l (3.4–20.5); and direct bilirubin (DBIL), 113.7 μmol/l (0.0–6.8). Coagulation function: prothrombin time (PT), 17.8 s (11.0–14.3); prothrombin time activity (PTA), 58% (80.0–120.0); and PT-international normalized ratio (PT-INR), 1.45 (0.90–1.10). Serological markers of hepatitis B: Hepatitis B virus surface antigen (HBsAg), > 250.00 IU/ml (0.00–0.05); Hepatitis B virus e antigen (HBeAg), 0.319 S/CO (negative) (< 1.00); Hepatitis B virus e antibody (HBeAb), 0.57 S/CO (positive) (> 1.00); Hepatitis B virus core antibody (HBcAb), 4.82 S/CO (positive) (< 1.00); HBcAb IgM, 35.20 S/CO (positive) (< 1.00); and HBV DNA, 2.12 × 10^7^ IU/ml. Abdominal CT revealed periportal exudation, gallbladder wall edema and thickening. The patient was diagnosed with “acute icteric hepatitis B”.

Considering the tendency of severe hepatitis, the patient was administered 0.5 mg ETV once per day, and atorvastatin was replaced with rosuvastatin. The HBV DNA level decreased to 1.38 × 10^3^ IU/mL after 10 days of treatment; however, the coagulation function continued to deteriorate within 5 days after admission, and bilirubin levels increased within 3 weeks after admission. Twenty-two days after admission, the patient began to suffer from muscle pain in both lower limbs. The results of laboratory examinations were as follows: creatine kinase (CK), 2503 U/L (26–192); lactate dehydrogenase (LDH), 380 U/L (135–225); and serum myoglobin, > 1200.00 ng/ml (0.00–116.30). Renal function was normal, as was Troponin I (TnI). Electroneurography showed that the motor conduction velocity (MCV) of the bilateral tibial nerve was slowed and the peripheral nerve was damaged. Due to the presence of a cardiac stent and abnormal coagulation function, the patient did not receive a lower extremity MR or muscle biopsy.

Due to the consideration of rhabdomyolysis, rosuvastatin was withdrawn; however, the patient’s symptoms did not improve, and CK increased to 4709 U/L (26–192). ETV was replaced with Tenofovir Alafenamide Fumarate (TAF) Tablets after 3 days. Following 3 days of TAF treatment, CK decreased to 4620 U/L (26–192), and the muscle pain symptoms gradually dissipated. The CK reduced to normal after rhabdomyolysis occurred for 13 days. Three months after disease onset, the liver function returned to normal and HBsAg was negative. TAF was discontinued 2 months after HBsAg became negative and atorvastatin was administered to reduce cardiovascular risk and mortality due to cardiovascular disease. After 1 year, no muscle pain or liver injury was observed.

## Discussion and conclusions

Rhabdomyolysis is a serious and potentially life threatening condition. A reasonable definition for the diagnosis of rhabdomyolysis includes the elevation of serum CK activity at least 10 times higher than the upper limit, followed by a rapid decrease of the sCK level to (near) normal levels. Acute renal failure due to acute tubular necrosis resulting from mechanical obstruction by myoglobin is the most common complication. Timely diagnosis of rhabdomyolysis is key for its treatment.

The patient had no myalgia at admission, and CK was normal. After 3 weeks, she experienced muscle weakness, myalgia and pigmenturia. Additionally, her serum CK and myoglobin levels increased progressively, and urine occult blood was positive. The diagnosis of rhabdomyolysis was clear. Many causes of rhabdomyolysis have been identified and can be categorised as acquired and inherited. In 75% of patients, the first episode of rhabdomyolysis is provoked by an acquired cause [1]. The most common acquired causes include substance abuse (34%), medication (11%), trauma (9%), and epileptic seizures (7%) [2]. Less frequent acquired causes include metabolic disturbances, infections, local muscle ischemia, generalized muscle ischemia, prolonged immobilization, exercise, and excessive heat [3]. In 60% of patients, there are two or more causative factors.

In this case, the patient had no muscle pain at admission, and the CK was normal. Thus, we considered that the presence of rhabdomyolysis was not related to atorvastatin usage, but might have been related to the use of rosuvastatin. The reason for altering the treatment to rosuvastatin was that rosuvastatin is not substantially metabolized by CYPs, and therefore, is not susceptible to drug-drug interactions. Rosuvastatin is known to be weakly metabolized by CYP2C9. In clinical trials of rosuvastatin, about three in 10,000 patients acquired severe myopathy [4]. Myopathy attributed to rosuvastatin occurs in ≤ 0.03% of patients receiving 10 to 40 mg of rosuvastatin and no cases of rhabdomyolysis have occurred in patients receiving 10 to 40 mg of rosuvastatin.

Statin-induced myopathy encompasses a heterogeneous group of muscle manifestations that have not been well characterized. Mitochondrial dysfunction, oxidative stress, and several mechanisms involved in impaired mevalonate metabolism, such as the isoprenylation of small G-proteins, have been implicated in statin toxicity [5].

The most typical adverse reactions of oral nucleoside/nucleotide analogues (NAs) are mitochondrial damage, including myotoxicity, nephrotoxicity and lactic acidosis. To date, there have been no case reports in English of ETV-induced rhabdomyolysis. Through a literature search, we found that Beijing Ditan Hospital Affiliated with the Capital Medical University in China reported that a patient with chronic hepatitis B developed rhabdomyolysis after treatment with 0.5 mg to 1.0 mg ETV. In our case, after stopping statin use for 3 days, the symptoms were not relieved and CK and LDH levels were further increased. Considering that the patient may have developed rhabdomyolysis from the combined treatment of ETV with statins, we replaced entecavir with TAF and achieved a good outcome.

Our case suggests that although ETV-induced rhabdomyolysis is extremely rare, clinicians should consider it in people taking statins. If patients have symptoms of myalgia, CK and myoglobin levels should be closely monitored. It should be noted that rhabdomyolysis may occur when nucleotide analogues are combined with lipid-lowering drugs, and patients with original myopathy should avoid taking the two kinds of drugs simultaneously to avoid the occurrence of rhabdomyolysis.

## Data Availability

Not applicable (no datasets were generated or analyzed during this study).

## Abbreviations

ETV: Entecavir
PCI: Percutaneous coronary intervention
ALT: Alanine aminotransferase
ALP: Alkaline phosphatase
GGT: Gamma glutamyltransferase
TBIL: Total bilirubin
DBIL: Direct bilirubin
PT: Prothrombin time
PTA: Prothrombin time activity
PT-INR: PT-international normalized ratio
HBsAg: Hepatitis B virus surface antigen
HBeAb: Hepatitis B virus e antibody
HBcAb: Hepatitis B virus core antibody
CK: Creatine kinase
LDH: Lactate dehydrogenase
TnI: Troponin I
MCV: Motor conduction velocity
TAF: Tenofovir Alafenamide Fumarate
